# Cell-death-associated inflammatory remodeling of the SPP1/KRT5 axis in high-grade serous ovarian cancer: multi-omics, single-cell, and irradiation validation

**DOI:** 10.3389/fcell.2026.1949173

**Published:** 2026-10-28

**Authors:** Jie Liu, Yingchun Zheng, Zhen Li, Ni Li

**Affiliations:** 1 Department of Reproductive Medicine, Qingdao Hospital, University of Health and Rehabilitation Sciences (Qingdao Municipal Hospital), Qingdao, China; 2 Department of Gynecologic Oncology, Qingdao Central Hospital, University of Health and Rehabilitation Sciences, Qingdao, China; 3 Department of Reproductive Medicine, Women and Children’s Hospital, Qingdao University, Qingdao, China

**Keywords:** high-grade serous ovarian cancer, inflammation, irradiation, KRT5, neoadjuvant chemotherapy, regulated cell death, single-cell RNA sequencing, SPP1

## Abstract

**Background:**

High-grade serous ovarian cancer (HGSOC) is characterized by extensive cellular heterogeneity and frequent emergence of chemotherapy resistance. Therapy-induced cellular injury and regulated cell death can reshape inflammatory signaling and the tumor microenvironment, but the relationship between death-associated stress responses and secreted phosphoprotein 1 (SPP1/osteopontin)-expressing myeloid cells or keratin 5 (KRT5)-positive epithelial states remains incompletely defined.

**Methods:**

The Cancer Genome Atlas Ovarian Cancer (TCGA-OV) raw RNA-seq counts and clinical annotations were analyzed in 429 tumor samples. Single-cell RNA sequencing data from GSE165897 included 22 paired treatment-naive and post-neoadjuvant chemotherapy (post-NACT) libraries from 11 patients with metastatic High-grade serous ovarian cancer (51,786 cells). GSE316118 provided RNA-seq profiles of KRT5+ and KRT5- SKOV3 populations (n = 3 per group). Differential expression, pathway enrichment, program scoring, paired sample-level comparisons, and cross-layer consistency analyses were performed. In addition, SK-OV-3 cells were exposed to 0 or 6 Gy X-ray irradiation and SPP1, HIF1A, VEGFA, and KRT5 were quantified by RT-qPCR. Regulated cell death was treated as a mechanistic context rather than directly assigned from transcriptomic data.

**Results:**

In TCGA-OV, SPP1 expression was most strongly associated with the inflammatory program (Spearman r = 0.63), with weaker positive associations with hypoxia (r = 0.42) and EMT (r = 0.35); SPP1-high versus SPP1-low groups did not differ significantly in overall survival (log-rank P = 0.181). At single-cell resolution, SPP1 localized predominantly to the myeloid compartment, whereas KRT5 expression was restricted to a subset of epithelial cells. After NACT, epithelial VIM expression and the epithelial hypoxia program increased significantly, while myeloid SPP1 showed a non-significant downward trend. In the SKOV3 dataset, KRT5+ and KRT5-populations showed reciprocal exploratory KRT5-CPC and SPP1-niche signatures. The positive SPP1-inflammatory coupling observed in TCGA-OV was reproduced at sample level in the independent single-cell cohort (r = 0.71). In irradiated SK-OV-3 cells, SPP1, HIF1A, VEGFA, and KRT5 were significantly increased at 24 h (all P < 0.01). Together, these findings are consistent with therapy-associated inflammatory remodeling in a cellular injury/death context, without establishing a specific cell-death modality.

**Conclusion:**

These data identify SPP1-marked myeloid inflammation and KRT5-associated epithelial states as complementary features of metastatic HGSOC and support a cell-death-associated inflammatory remodeling framework linking treatment stress with epithelial hypoxia/mesenchymal programs. The findings are associative and hypothesis-generating; specific regulated cell-death pathways require direct functional validation.

## 1 Introduction

High-grade serous ovarian cancer (HGSOC) accounts for most ovarian cancer deaths and is commonly diagnosed at an advanced stage. Although cytoreductive surgery and platinum-taxane chemotherapy can produce substantial initial responses, recurrent disease and acquired chemoresistance remain major causes of treatment failure (Sung et al., 2021; Lheureux et al., 2019). Molecular heterogeneity within malignant cells and the surrounding tumor microenvironment complicates the identification of stable biomarkers and treatment-associated cellular states.

The tumor microenvironment contains interacting epithelial, immune, stromal, and endothelial compartments that can influence treatment response and disease progression (Binnewies et al., 2018; Qian et al., 2020). Among myeloid populations, SPP1-expressing tumor-associated macrophages have emerged as a recurrent inflammatory and immunoregulatory state across cancers and have also been implicated in ovarian cancer through the SPP1-CD44 axis (Hou et al., 2025; Wang et al., 2025). In parallel, recent work in HGSOC has identified KRT5-positive cancer-propagating cells and an osteopontin-producing KRT5-negative niche, providing a biologically relevant framework for examining the relationship between KRT5-associated epithelial states and SPP1-associated microenvironmental programs (Bidarimath et al., 2026).

Single-cell RNA sequencing (scRNA-seq) enables these cellular programs to be resolved at lineage and subtype level rather than inferred from bulk averages (Qian et al., 2020; Hwang et al., 2018). The GSE165897 cohort provides paired treatment-naive and post-neoadjuvant chemotherapy (post-NACT) samples from 11 patients with metastatic HGSOC and has previously shown chemotherapy-associated stress-state remodeling at single-cell resolution (Zhang et al., 2022). TCGA-OV provides an independent bulk-transcriptomic context for examining cohort-level SPP1-associated programs and clinical associations (Cancer Genome Atlas Research Network, 2011).

Regulated cell death (RCD) provides a mechanistic bridge between anticancer treatment and inflammatory tissue remodeling. Apoptosis, necroptosis, pyroptosis, and ferroptosis differ in their execution mechanisms and immunologic consequences, while lytic or immunogenic death can release damage-associated molecular patterns (DAMPs) and other stress signals that recruit or reprogram immune and stromal cells (Galluzzi et al., 2018; Galluzzi et al., 2017). In tumors, these signals may couple treatment-induced injury to inflammatory, hypoxic, and reparative microenvironmental states. However, transcriptomic associations alone cannot determine whether a specific RCD modality is active; direct biochemical or functional measurements are required.

In the present study, we integrated TCGA-OV bulk transcriptomics, paired single-cell profiling from GSE165897, and the KRT5-sorted SKOV3 dataset GSE316118. We aimed to (i) define transcriptome-wide programs associated with SPP1 in bulk ovarian tumors; (ii) localize SPP1 and KRT5-associated states across epithelial, immune, and stromal lineages; (iii) quantify paired pre/post-NACT changes at sample level; and (iv) compare these treatment-associated effects with the KRT5+/KRT5-cell-state axis. We therefore asked whether the SPP1/KRT5 axis and treatment-associated hypoxia/EMT programs could be interpreted within a broader cell-death-associated inflammatory remodeling framework. Because ionizing radiation is a well-characterized exogenous stressor capable of inducing cellular injury/death together with hypoxia-linked and epithelial-mesenchymal transition (EMT)-associated transcriptional responses in cancer cells, we additionally used irradiated SK-OV-3 cells as an orthogonal *in vitro* stress model for RT-qPCR assessment of selected bioinformatically prioritized genes (Barker et al., 2015; Dewhirst et al., 2008; Barcellos-Hoff et al., 2005; Kalluri and Weinberg, 2009; Nieto et al., 2016; Zhang et al., 2011; Zhou et al., 2011). This design was intended to identify reproducible cross-layer relationships without inferring specific death modalities or causal mechanisms not directly measured.

## 2 Materials and methods

### 2.1 Datasets

Three public datasets were used in human ovarian serous carcinoma/HGSOC-relevant contexts (dataset audit in Data/dataset_audit.md): (1) TCGA-OV raw STAR counts and clinical annotations for 429 TCGA-OV tumor samples (421 primary, 8 recurrent); tumor grade was not reported for any sample in the local clinical table (GDC Data Portal); (2) GSE165897, 10x Genomics UMI counts with official cell-type annotations for 22 metastatic HGSOC libraries from 11 patients sampled before (treatment-naive) and after neoadjuvant chemotherapy (post-NACT) [51,786 cells] (Zhang et al., 2022); and (3) GSE316118, 3’ QuantSeq RNA-seq of SKOV3 cells sorted by KRT5 promoter activity (KRT5 + n = 3, KRT5-n = 3; deposited as normalized counts) (Bidarimath et al., 2026). GSE185386, cited in an earlier version of this work as post-radiotherapy HGSOC single-cell data, is officially a melanoma brain-metastasis atlas and was excluded after direct GEO verification.

### 2.2 Cell-death-associated interpretive framework

The public transcriptomic datasets do not contain direct biochemical measurements of regulated cell-death execution. Accordingly, ferroptosis, pyroptosis, necroptosis, and apoptosis were not assigned to individual cells or samples from gene expression alone. In this manuscript, “cell-death-associated” refers to the treatment-injury context in which death and stress responses can release inflammatory signals and remodel the microenvironment (Galluzzi et al., 2018; Galluzzi et al., 2017; Barker et al., 2015; Dewhirst et al., 2008; Barcellos-Hoff et al., 2005; Kalluri and Weinberg, 2009; Nieto et al., 2016; Zhang et al., 2011; Zhou et al., 2011). The measured endpoints remain transcriptomic programs, lineage-specific expression patterns, and irradiation-responsive RT-qPCR candidates.

### 2.3 Bulk transcriptome analysis (TCGA-OV)

The TCGA-OV cohort comprised 429 TCGA-OV tumor samples (421 primary, 8 recurrent); tumor grade was not reported for any record in the local clinical table. Genes with at least 10 counts in at least 10% of samples were retained (17,225 genes). Counts were converted to log2(CPM+1) for visualization, scoring and correlation. SPP1-high vs. SPP1-low groups were defined by the median of SPP1 log2(CPM+1). Differential expression used DESeq2 (pydeseq2 0.5.4, Wald test, BH-adjusted) (Muzellec et al., 2023). Preranked GSEA (gseapy 1.2.1; KEGG_2021_Human and MSigDB_Hallmark_2020; 1,000 permutations) and Enrichr ORA (KEGG_2021_Human, GO_Biological_Process_2021, MSigDB_Hallmark_2020) annotated SPP1-associated programs (Fang et al., 2023). Six programs were scored per sample as the mean z-score of member genes: KRT5_CPC_UP and SPP1_NICHE_UP (defined from GSE316118, below) and Hallmark EMT, HYPOXIA, INFLAMMATORY_RESPONSE and E2F_TARGETS. Overall survival (OS) used days-to-death/death events and days-to-last-follow-up for censoring; Kaplan-Meier curves with log-rank tests and univariate Cox models (lifelines 0.30.3) compared median-split groups and standardized continuous scores. FIGO stage association used Kruskal-Wallis tests.

### 2.4 Single-cell analysis (GSE165897)

The deposited UMI matrix and cell annotations were analyzed with Scanpy 1.12.2 (Wolf et al., 2018). Light quality filters (detected genes ≥200, mitochondrial fraction <20%) retained all 51,786 cells. Counts were normalized to 10,000 per cell and log1p-transformed; 3,000 highly variable genes (cell_ranger flavor), 50 principal components, a k = 15 neighbor graph, UMAP and Leiden clustering (resolution 0.5) were computed with a fixed seed. Official EOC/Stromal/Immune labels were retained; immune cells were refined to T, NK, Myeloid, B, Plasma and Mast lineages and stromal cells to Fibroblast vs. Endothelial by mean canonical-marker expression (marker panels in config. py). Six program scores were computed with scanpy score_genes using expression-matched control genes. Composition was computed per library; pseudobulk profiles were aggregated from raw UMI counts per sample x lineage (≥10 cells). Paired comparisons (post-NACT vs. treatment-naive, n = 11 pairs) used paired t-tests on log2(CPM+1) pseudobulk and paired Wilcoxon tests on sample-level aggregates; cell-level distributions are shown descriptively only.

### 2.5 SKOV3 sorted-population analysis (GSE316118)

Because the deposited matrix contains normalized (non-integer) counts, genes with value > 1 in at least 2 of 6 libraries were log2 (x+1) transformed and tested with limma-style moderated t-statistics (empirical-Bayes variance moderation, BH correction) (Smyth, 2004). The two project signatures KRT5_CPC_UP (up in KRT5+) and SPP1_NICHE_UP (up in KRT5-) were defined at nominal p < 0.01 (exploratory; n = 3 per group) with |log2FC|>1 and capped at 200 genes per direction; because the definition contrast has n = 3 and three per group they are labeled exploratory throughout. Signature scores are mean z-scores of member genes.

### 2.6 *In vitro* cell culture, irradiation and RT-qPCR validation

Human SK-OV-3 (SKOV3) ovarian adenocarcinoma cells were obtained from the American Type Culture Collection (ATCC, HTB-77). Cells were maintained in McCoy’s 5A modified medium supplemented with 10% fetal bovine serum and 1% penicillin-streptomycin at 37 °C in a humidified atmosphere containing 5% CO2, consistent with ATCC recommendations and published SKOV3 culture conditions (Bidarimath et al., 2026). Cells in logarithmic growth were seeded 24 h before irradiation.

For the *in vitro* irradiation experiment, cultures were assigned to sham irradiation (0 Gy) or a single 6-Gy ionizing-radiation exposure delivered with a calibrated 6-MV X-ray source at approximately 2 Gy/min. Sham controls were handled identically without beam delivery. Cells were returned to standard culture conditions immediately after irradiation and harvested 24 h later for RNA analysis. This design was used to assess acute transcriptional stress responses rather than to infer clonogenic radiosensitivity. Radiation-induced remodeling of hypoxia-related signaling and EMT-associated programs has been well documented in tumor models (Barker et al., 2015; Dewhirst et al., 2008; Barcellos-Hoff et al., 2005; Kalluri and Weinberg, 2009; Nieto et al., 2016; Zhang et al., 2011; Zhou et al., 2011).

Total RNA was isolated using a phenol/guanidinium-based RNA extraction reagent, quantified spectrophotometrically, and reverse-transcribed using equal RNA input for each sample. Real-time quantitative PCR (RT-qPCR) was performed using SYBR Green chemistry. For the four-gene validation presented in Figure 1, candidate transcripts were SPP1, HIF1A, VEGFA, and KRT5; GAPDH was used as the endogenous reference gene. Each biological sample was analyzed in technical triplicate. Relative transcript abundance was calculated by the 2^-ΔΔCt^ method with the 0-Gy group as the calibrator, following MIQE reporting principles (Bustin et al., 2009; Livak and Schmittgen, 2001). Three independent biological experiments were performed for the validation analysis; data are summarized as mean ± SD, and 0-Gy versus 6-Gy comparisons were assessed using two-sided Student t-tests with P < 0.05 considered statistically significant.

**FIGURE 1:**
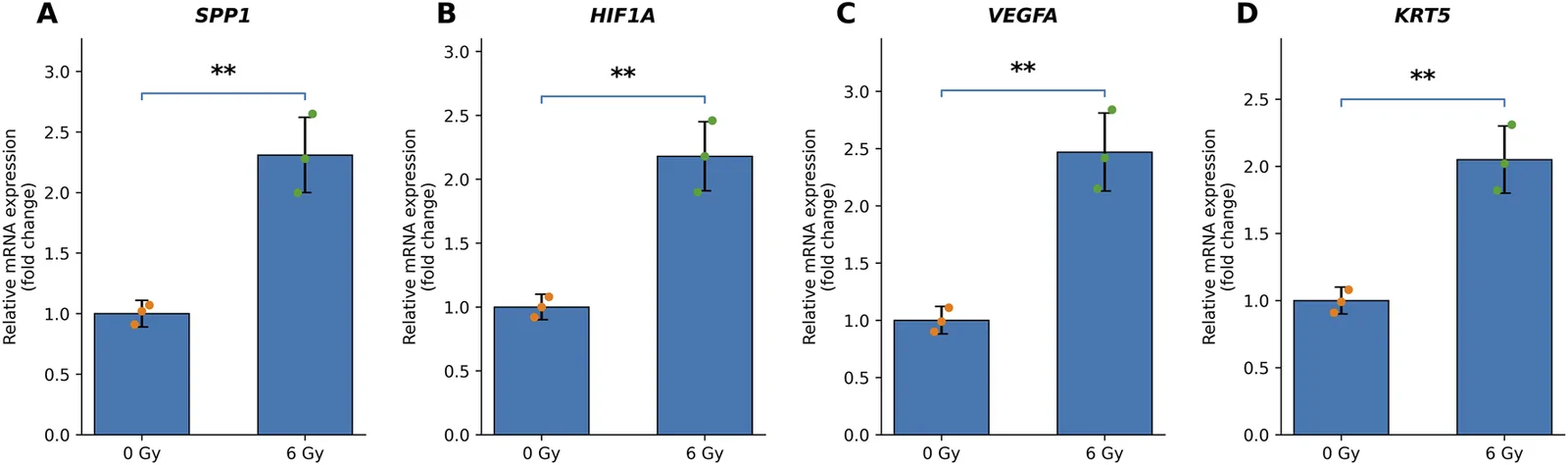
RT-qPCR validation of cell-death-associated stress-response candidates after *in vitro* irradiation in SK-OV-3 cells. SK-OV-3 cells (ATCC HTB-77) were exposed to 0 Gy (control) or 6 Gy X-ray irradiation. Total RNA was collected 24 h after irradiation, and relative mRNA expression of **(A)** SPP1, **(B)** HIF1A, **(C)** VEGFA, and **(D)** KRT5 was determined by RT-qPCR. Expression was normalized to GAPDH and calculated using the 2^-ΔΔCt method. Bars show mean ± SD from three independent biological experiments; **P < 0.01 versus the 0-Gy control (two-sided Student’s t-test).

### 2.7 Software and reproducibility

Python 3.12.5 with pandas 3.0.3, numpy 2.4.6, scipy 1.17.1, scanpy 1.12.2, anndata, pydeseq2 0.5.4, gseapy 1.2.1, lifelines 0.30.3, statsmodels 0.14.6, scikit-learn 1.9.0, matplotlib 3.11.0. All thresholds are maintained in a single configuration file; input files are SHA-256-hashed (Data/input_hashes.tsv); analysis code is provided in Code/ (steps 00-08). Random seed 20250820 was used for all stochastic steps. RT-qPCR group comparisons were performed with SciPy 1.17.1 using the biological replicate as the unit of analysis.

## 3 Results

### 3.1 SPP1 marks an inflammatory program in the TCGA-OV cohort

QC hexbin of 429 TCGA-OV tumor samples (421 primary, 8 recurrent) showed library sizes centered near 6.2e+07 raw counts with 17,634 detected genes per sample (Figure 2A). PCA of the top 2,000 variable genes showed that SPP1-high and SPP1-low tumors separated along PC1 (Spearman r (PC1, SPP1) = 0.63, P = 1.0e-49; Figure 2B), consistent with the SPP1-associated inflammatory program being a principal axis of transcriptomic variation in the cohort. Pairwise sample correlation on the top 500 variable genes showed a continuous correlation structure without outlier samples (Figure 2C). SPP1 expression varied continuously across the cohort (median log2(CPM+1) = 8.34), and the median-split threshold defined SPP1-high (n = 215) and SPP1-low (n = 214) groups for downstream analysis (Figure 2D). SPP1 expression co-varied most strongly with the inflammatory program (r = 0.63, P = 1.3e-49), followed by hypoxia (r = 0.42) and EMT (r = 0.35), but not with the KRT5 cancer-propagating-cell program (r = −0.03, P = 0.52; Figure 2E). Kaplan-Meier analysis showed no significant overall survival difference between SPP1-high and SPP1-low patients (log-rank P = 0.181; median 1,364 vs. 1,355 days; Figure 2F).

**FIGURE 2:**
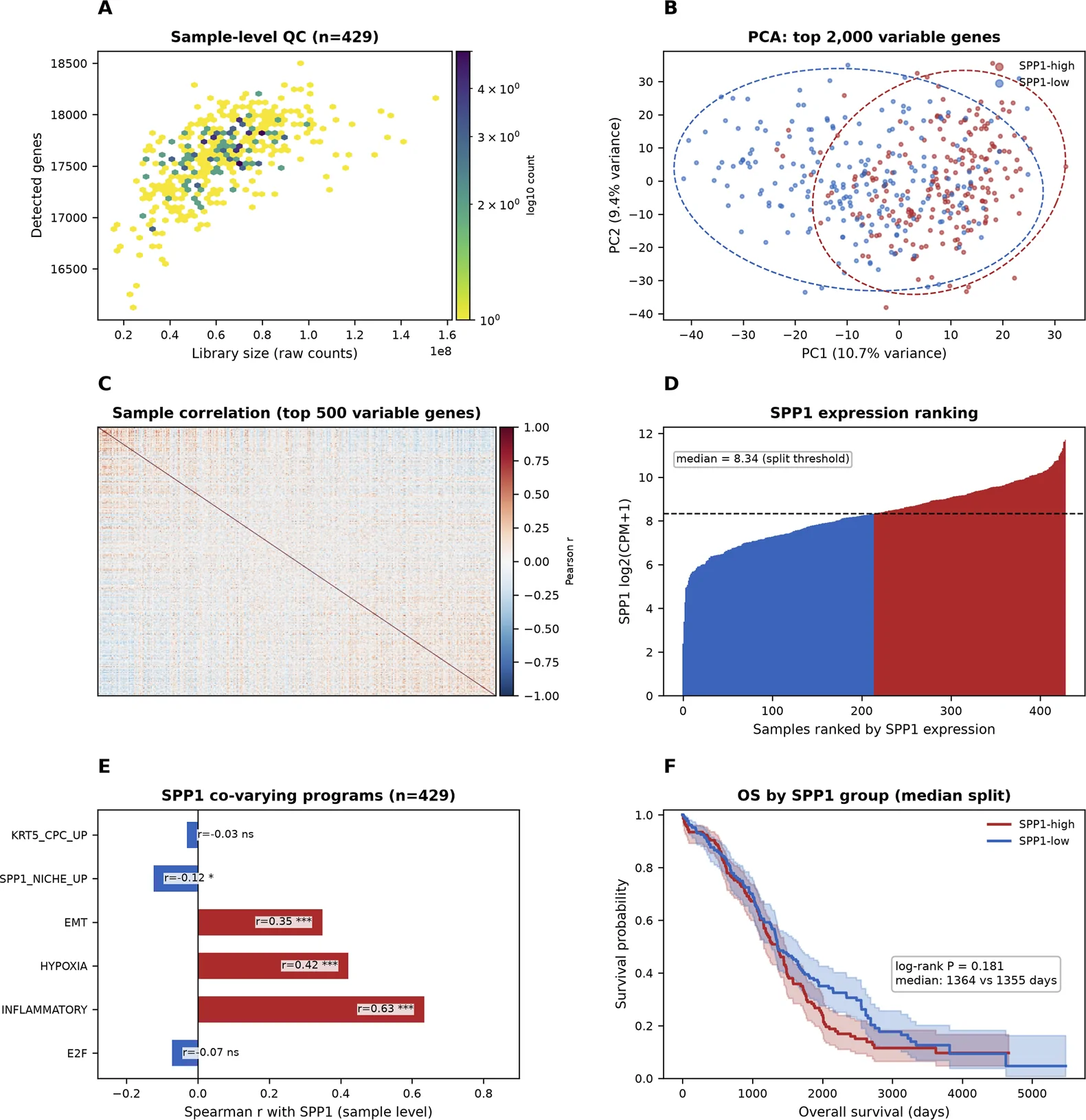
**(A)** Hexbin density of library size versus detected genes for the 429 TCGA-OV tumor samples (421 primary, 8 recurrent); color encodes log10 sample count. **(B)** PCA (top 2,000 variable genes, z-scored log2(CPM+1)) with samples colored by SPP1-high/SPP1-low; dashed ellipses are 95% data ellipses. **(C)** Pearson sample correlation heatmap (top 500 variable genes); samples ordered by increasing SPP1 expression. **(D)** Ranked SPP1 expression (log2(CPM+1)) for all samples; dashed line marks the median (8.34) used to define SPP1-high/SPP1-low groups. **(E)** Spearman correlations between SPP1 expression and six program scores across all 429 TCGA-OV tumor samples (421 primary, 8 recurrent); stars denote nominal P < 0.05/0.01/0.001. **(F)** Kaplan-Meier overall survival by SPP1 group (median split); ticks mark censored patients; log-rank P and group medians annotated.

## 3.2 Transcriptome-wide SPP1-associated programs in bulk tumors

DESeq2 contrasting SPP1-high versus SPP1-low tumors identified 319 upregulated and 494 downregulated genes (FDR<0.05, |log2FC|>1; Figure 3A); SPP1 itself was the defining upregulated transcript, with MMP9 and CD44 among the top co-regulated genes. Preranked GSEA showed that SPP1-high tumors were enriched for inflammatory and immune hallmark programs, including inflammatory response, interferon-gamma response, allograft rejection, TNF-alpha signaling and complement (Figure 3B). Gene-ontology analysis of SPP1-high upregulated genes returned myeloid and inflammation terms dominated by neutrophil degranulation, cytokine-mediated signaling, inflammatory response and immune activation processes (Figure 3C). Along the SPP1 expression gradient, the inflammatory program tracked SPP1 closely, with EMT and hypoxia rising toward the SPP1-high end (Figure 3D), consistent with the correlations in Figure 2E. Among FDR-significant genes, the largest SPP1-associated increases included matrix, inflammatory and immune genes, while the strongest decreases involved epithelial and mitochondrial transcripts (Figure 3E). SPP1 expression alone did not vary with FIGO stage (P = 0.88), whereas the KRT5 cancer-propagating-cell program (P = 0.005) and the SPP1 niche program (P = 0.001) differed among stages. Both had higher median scores in stage II than stage IV (KRT5 CPC 0.08 vs. −0.06; SPP1 niche 0.14 vs. −0.06; Figure 3F).

**FIGURE 3:**
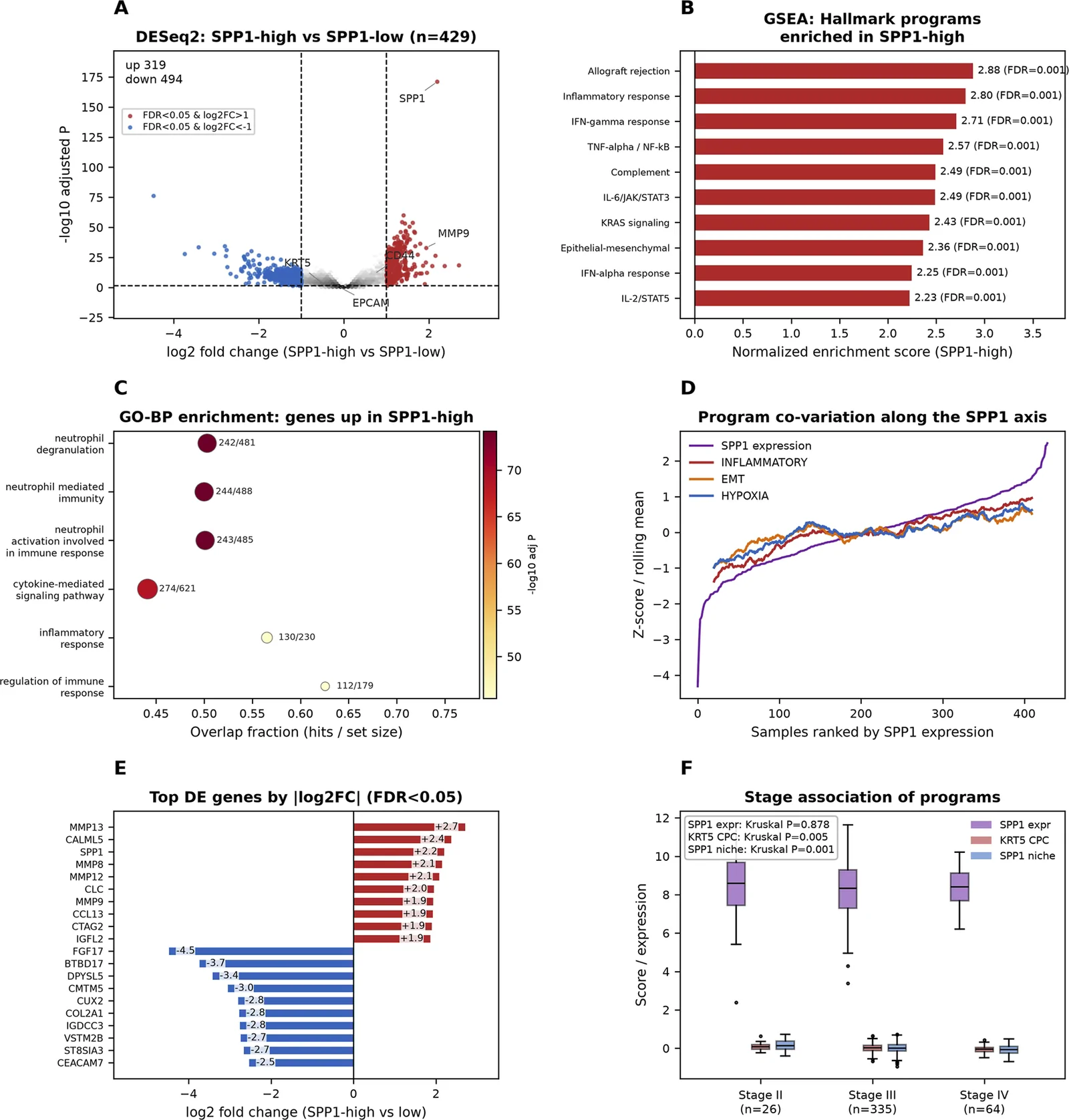
**(A)** Hexbin volcano of DESeq2 results (SPP1-high vs. SPP1-low); red/blue points are FDR<0.05 with |log2FC|>1; dashed lines mark FDR = 0.05 and log2FC = ±1 thresholds. **(B)** Top 10 Hallmark gene sets by |NES| from preranked GSEA; positive NES = enriched in SPP1-high tumors; FDR q-values annotated. **(C)** Top 6 GO-BP terms (Enrichr 2021) for SPP1-high upregulated genes; bubble size = overlapping genes, color = -log10 adjusted P. **(D)** Samples ranked by SPP1 expression (violet); rolling means (window = 40) of inflammatory (red), EMT (orange) and hypoxia (blue) scores. **(E)** Diverging bars for the 10 largest up- and 10 largest downregulated FDR-significant genes (SPP1-high vs. SPP1-low). **(F)** SPP1 expression (violet), KRT5 CPC program (red) and SPP1 niche program (blue) by FIGO stage II/III/IV; Kruskal–Wallis P annotated.

### 3.3 Single-cell atlas of paired pre/post-NACT metastatic HGSOC

Per-library distributions of detected genes were broadly comparable across the 22 treatment-naive and post-NACT libraries (Figure 4A). The UMI-gene relationship remained continuous with a single dense population and no low-quality tail (Figure 4B). Mitochondrial fractions were low in both phases and the majority of cells fell below the 20.0% cutoff (Figure 4C). The UMAP atlas recapitulated the three official compartments (EOC, Stromal, Immune) with well-separated neighborhoods (Figure 4D). Marker-based refinement of the official annotation resolved nine major lineages, with T cells the most abundant immune population (Figure 4E). Cells from treatment-naive and post-NACT specimens intermingled in the shared embedding, indicating that phase differences are graded rather than batch-like (Figure 4F).

**FIGURE 4:**
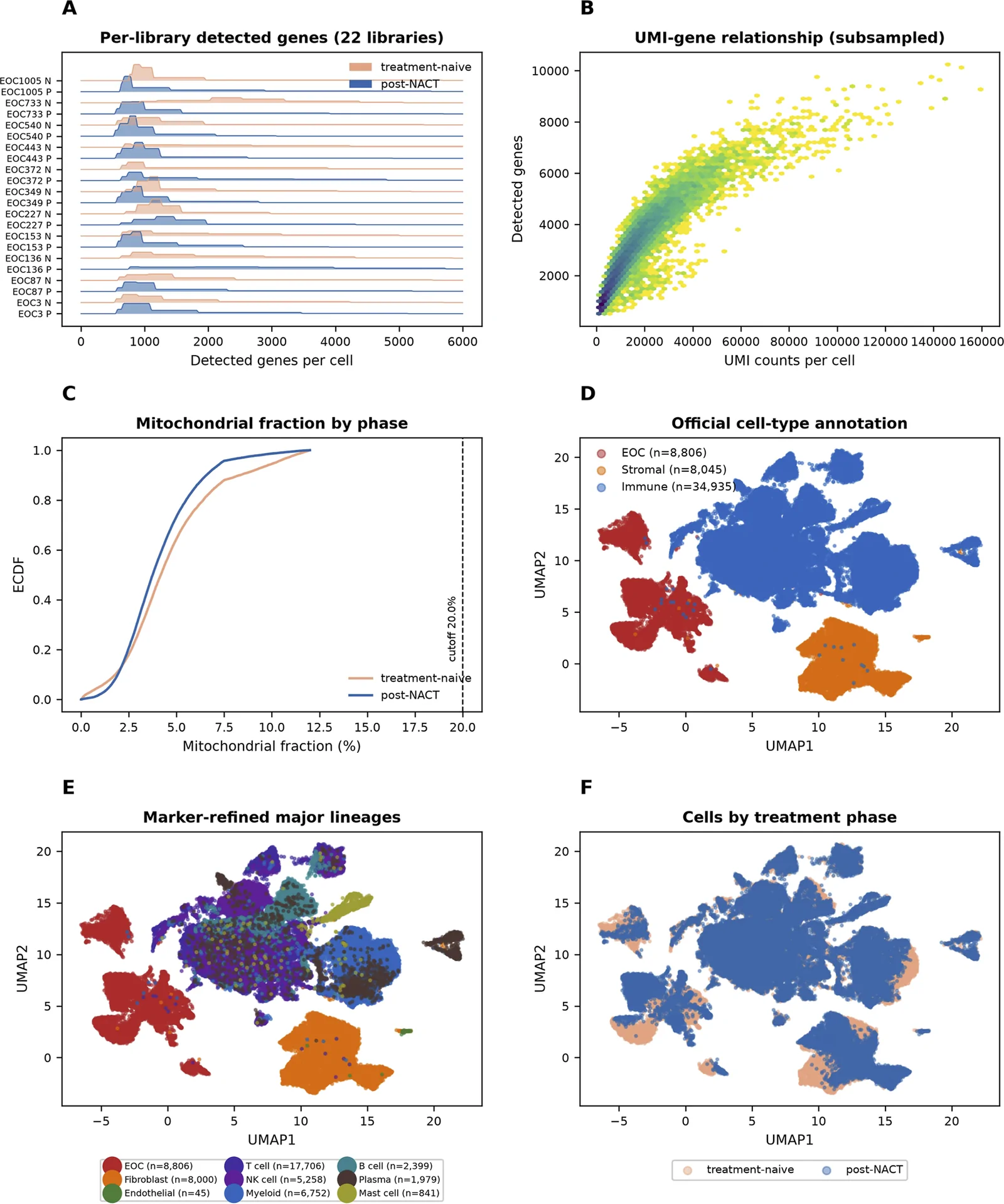
**(A)** Detected-gene ridgelines per library (quantile-interpolated density), colored by treatment phase. **(B)** Hexbin density of UMI counts versus detected genes; color encodes log10 cell counts (display subsample 1,500 cells per library). **(C)** ECDF of mitochondrial fraction by treatment phase; dashed line marks the 20.0% cutoff. **(D)** UMAP colored by the official cell-type labels provided with GSE165897; cell counts annotated. **(E)** UMAP colored by marker-refined major lineages; legend lists lineage cell counts. **(F)** UMAP colored by treatment phase (treatment-naive vs. post-NACT).

### 3.4 Annotation and composition of the metastatic microenvironment

Each refined lineage showed the expected canonical marker pattern (EPCAM/PAX8 in EOC, COL1A1/DCN in fibroblasts, PECAM1 in endothelium, CD3D in T-cell, CD14/C1QA in myeloid cells; Figure 5A). T cells, EOC and fibroblasts dominated the libraries with substantial inter-patient heterogeneity in composition (Figure 5B). Within-patient comparison showed post-NACT upward trends for T-cell (median delta +0.093; 9/11 patients) and NK-cell fractions (+0.015; 7/11 patients) with a variable EOC change (median delta +0.024); none of these compositional shifts reached paired significance (all P > 0.17, Figures 5C, 6A). At subtype resolution, macrophages remained the largest myeloid population while T Cell subtypes were consistently abundant in both phases (Figure 5D). EOC cells carried the highest median UMI and detected-gene counts, followed by fibroblast and endothelial lineages; myeloid cells were intermediate on both measures (Figure 5E). The marker evidence matrix confirmed lineage-specific marker blocks with no off-diagonal cross-reactivity (Figure 5F).

**FIGURE 5:**
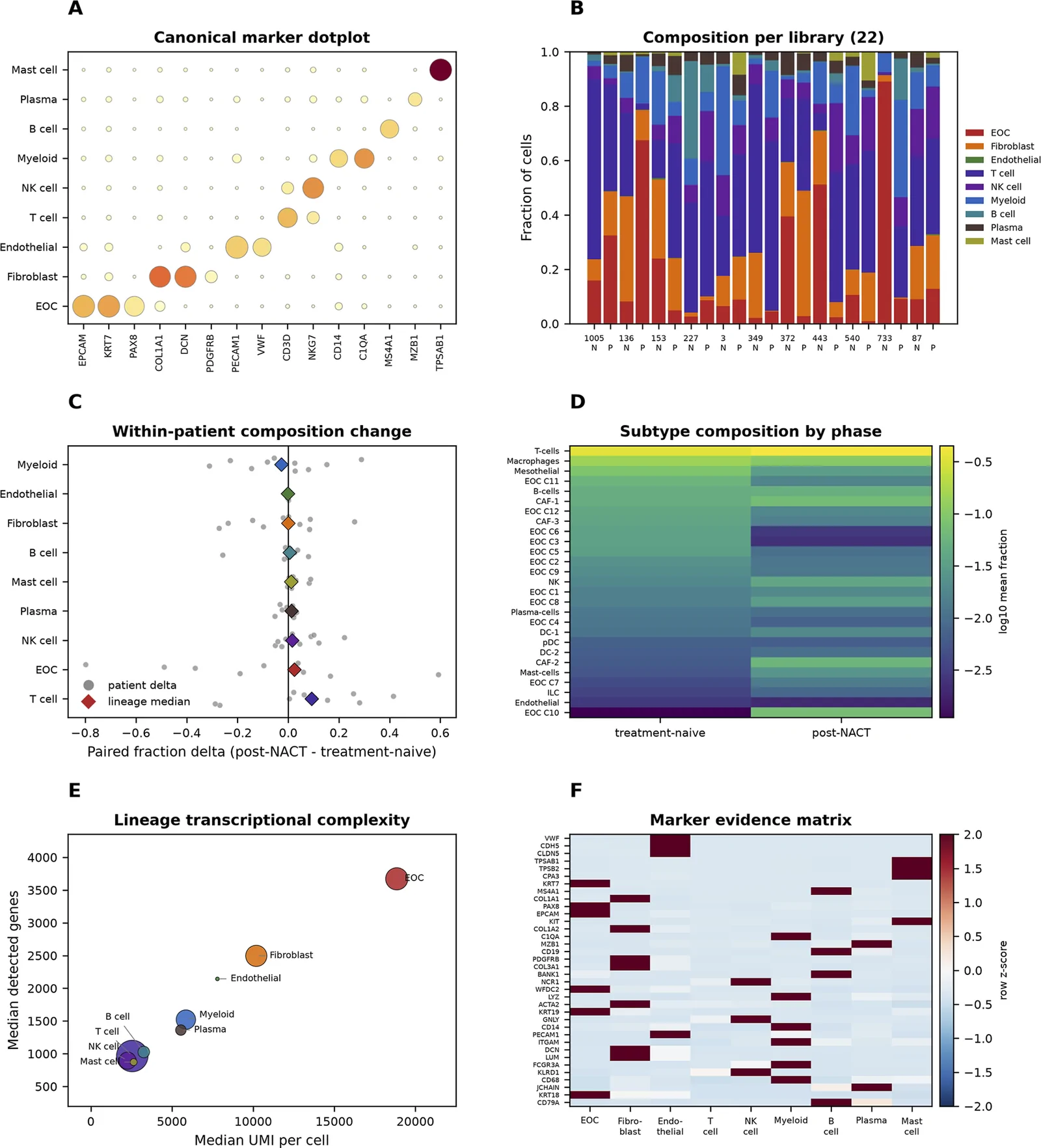
**(A)** Dotplot of canonical markers across major lineages; dot size = fraction expressing, color = mean log1p expression. **(B)** Stacked lineage composition of each library; libraries ordered by patient with treatment-naive before post-NACT. **(C)** Paired post-NACT-minus-naive lineage-fraction deltas for each patient (gray circles); diamonds mark lineage medians. **(D)** Heatmap of log10 mean subtype fractions in treatment-naive vs. post-NACT libraries. **(E)** Median UMI vs. detected genes per lineage; bubble size scales with cell number. **(F)** Row-z-scored mean expression of the 36 most lineage-informative canonical markers across lineages (z-score for display only).

**FIGURE 6:**
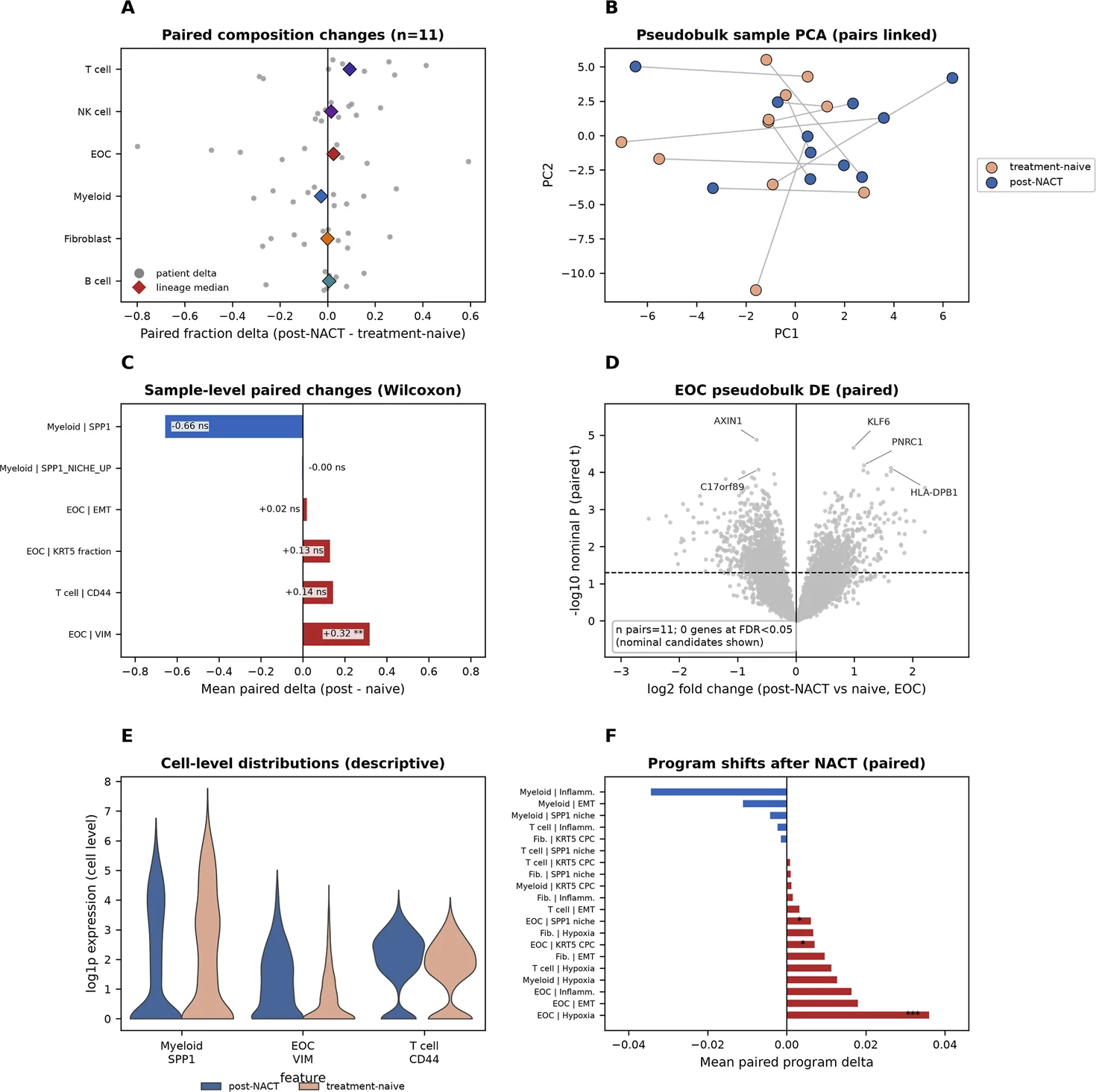
**(A)** Paired post-NACT-minus-naive lineage-fraction deltas for each patient (gray circles); diamonds mark lineage medians. **(B)** PCA of sample-level pseudobulk profiles (top 1,000 variable genes); gray lines connect paired samples of the same patient. **(C)** Mean paired post-minus-naive deltas for preset lineage-feature pairs; stars denote paired Wilcoxon P < 0.05/0.01/0.001. **(D)** Volcano of paired EOC pseudobulk tests (post-NACT vs. naive); dashed line marks nominal P = 0.05; top nominal genes labeled; no gene passed BH FDR<0.05. **(E)** Cell-level log1p expression distributions (Myeloid SPP1, EOC VIM, T Cell CD44) by phase; descriptive display, inference is sample-level (panel C). **(F)** Mean paired post-minus-naive program-score deltas per lineage; stars denote paired Wilcoxon P < 0.05/0.01/0.001.

### 3.5 The SPP1/KRT5 axis at single-cell resolution

SPP1 expression localized to a discrete myeloid-enriched region of the atlas (13.6% of all cells; Figure 7A). KRT5 expression was restricted to a subset of epithelial cells (7.1% of all cells; Figure 7B), consistent with the cancer-propagating-cell marker model. The SKOV3-derived KRT5 cancer-propagating-cell program scored highest in myeloid cells (median +0.044) and lowest in EOC cells (−0.050), indicating that in patient tumors this cell-line-derived program is dominated by myeloid-expressed component genes; KRT5 expression itself remains epithelial-restricted (Figure 7B; see also Figure 7C). The subtype-level program map separated macrophage and dendritic subtypes (inflammatory) from CAF subtypes (EMT) and EOC subtypes (KRT5 program), recapitulating the compartment architecture (Figure 7D). Myeloid cells contributed 59% of all SPP1+ cells and 62% of myeloid cells expressed SPP1, identifying myeloid cells as the dominant osteopontin source in metastatic HGSOC (Figure 7E). Among EOC subtypes, EOC_C7 combined the highest KRT5 expression with the highest EMT program score among epithelial subtypes, with EOC_C6 second in KRT5 (Figure 7F), nominating them as KRT5+ cancer-propagating candidates in patient tumors.

**FIGURE 7:**
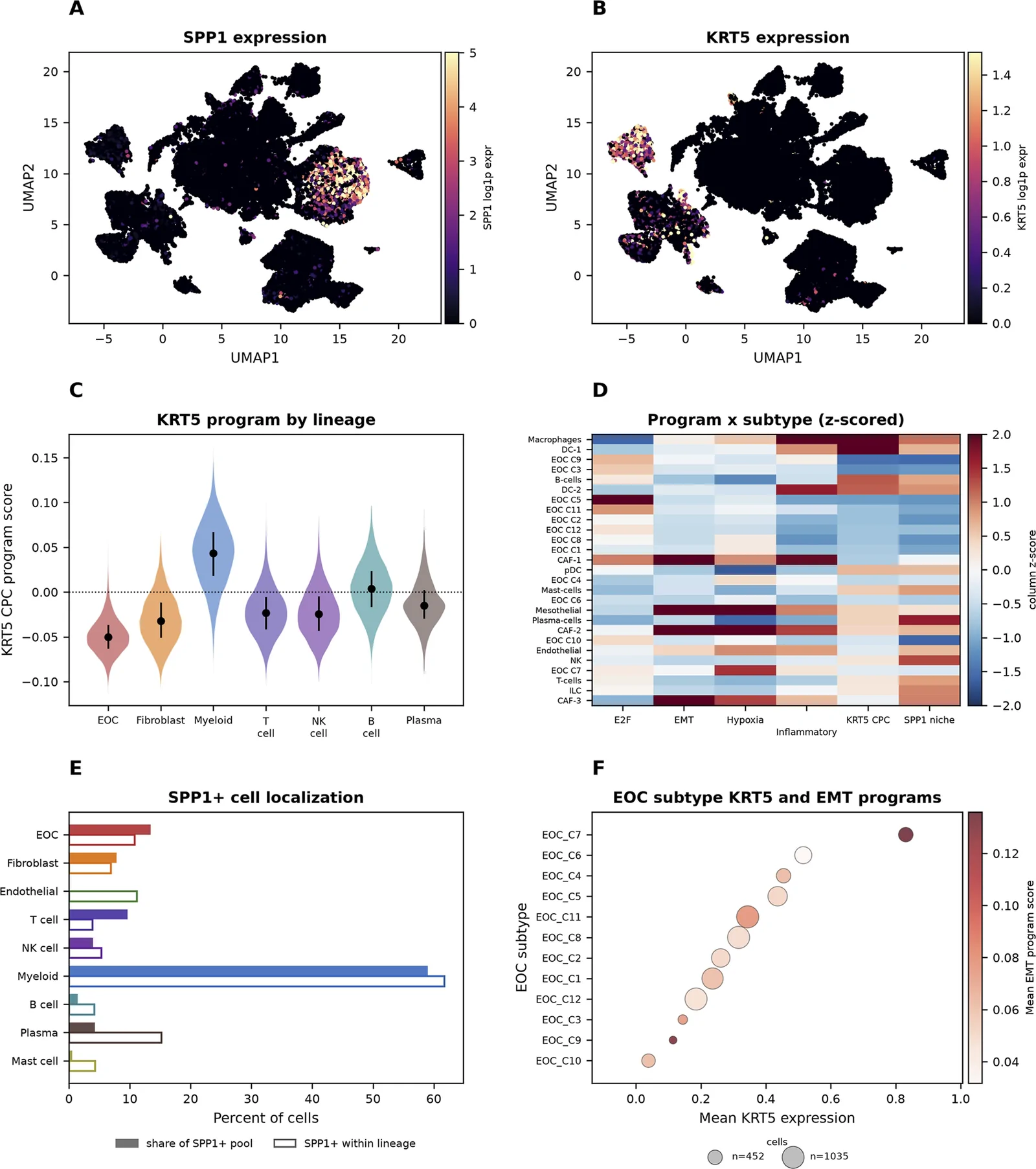
**(A)** UMAP colored by SPP1 log1p expression (color bar capped at the 99th percentile). **(B)** UMAP colored by KRT5 log1p expression (color bar capped at the 99th percentile). **(C)** KRT5 CPC program scores per lineage; violins show the cell-level distribution, markers show median (dot) and IQR (line). **(D)** Mean program scores per official subtype, column z-scored for display; rows ordered by the KRT5 program. **(E)** Paired horizontal bars show each lineage’s contribution to the SPP1+ pool (filled) and the fraction of that lineage expressing SPP1 (outlined). **(F)** EOC subtypes ranked by mean KRT5 expression; bubble color = mean EMT program score and bubble size = cell count.

### 3.6 Post-NACT remodeling of the microenvironment

Within-patient deltas showed upward T Cell trends in most patients and heterogeneous EOC changes after NACT (Figure 6A). Sample-level pseudobulk PCA did not separate phases globally, with paired patients moving in patient-specific directions (Figure 6B). At sample level, myeloid SPP1 expression trended lower after NACT (mean paired delta −0.66, P = 0.102) while EOC VIM increased significantly (delta +0.32, P = 0.00293; Figure 6C). Paired EOC pseudobulk testing identified no genes surviving transcriptome-wide FDR correction (0/14,966 at FDR<0.05; n = 11 pairs), so post-NACT EOC changes are reported descriptively from the nominal candidates (Figure 6D). Cell-level distributions mirrored the sample-level analysis: myeloid SPP1 shifted downward while EOC VIM and T Cell CD44 shifted upward after NACT (Figure 6E). Paired program deltas showed a significant post-NACT increase of the hypoxia program in EOC cells (P = 0.000977) together with smaller increases of the KRT5 CPC and SPP1 niche programs (P = 0.0186 and P = 0.0244), while the myeloid inflammatory program trended lower (P = 0.083; Figure 6F).

### 3.7 The KRT5^+/−^ niche model in SKOV3 sorted populations

PCA of the six SKOV3 libraries showed overlapping KRT5+ and KRT5-populations in the first two principal components (Figure 8A), indicating that their transcriptional differences are modest relative to replicate variability, consistent with the limited number of FDR-significant genes (panel B). Moderated t-testing identified 13 FDR-significant genes between KRT5+ and KRT5-populations (Figure 8B); SPP1 trended higher in the KRT5-niche fraction (log2FC = −0.74, nominal P = 0.11), consistent with the published osteopontin-producing niche model. The KRT5 cancer-propagating-cell program and the SPP1 niche program showed reciprocal enrichment across the sorted populations (Figure 8C); this separation is definitional because the programs were derived from the same contrast and is not presented as independent validation. Consistent with the published CPC-niche hierarchy, SPP1 was higher in KRT5- niche cells while CD44 and CCNE1 trended higher in KRT5+ cancer-propagating cells (Figure 8D). No KEGG term passed adjusted P < 0.05 for either derived program (Figure 8E); the highest-ranked terms were Synthesis and degradation of ketone bodies and Butanoate metabolism. Effect estimates confirmed the direction of the SPP1 niche axis: SPP1 was lower in KRT5+ CPCs (−0.74) while the KRT5+ program genes (e.g., KIRREL3, +3.81) were higher, although only a minority of axis genes reached FDR significance (Figure 8F).

**FIGURE 8:**
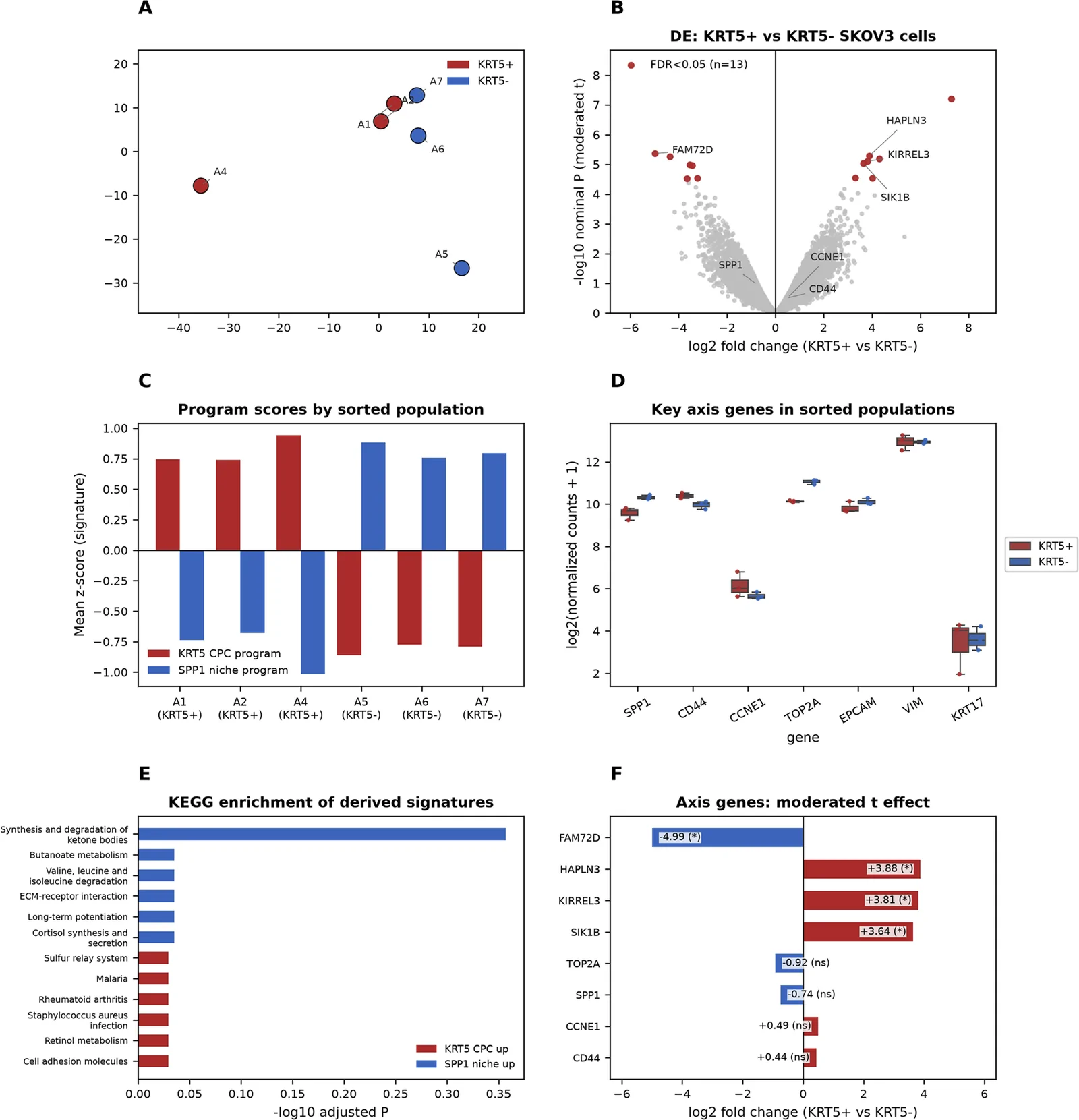
**(A)** PCA of the six SKOV3 libraries (top 1,000 variable genes, z-scored log2 (x+1)); KRT5+ red, KRT5-blue; sample labels annotated. **(B)** Volcano of moderated t-test results (KRT5+ vs. KRT5-); red points pass BH FDR<0.05 and selected genes are labeled. **(C)** Mean z-scores of the KRT5 CPC program (red) and SPP1 niche program (blue) per library; derived from this contrast (nominal P < 0.01, |log2FC|>1). **(D)** Expression of preset axis genes (log2 (x+1)) in KRT5+ vs. KRT5-libraries; points are individual libraries (n = 3 per group). **(E)** Six highest-ranked KEGG 2021 terms per derived signature; bars show -log10 adjusted P (0/60 tested terms passed adjusted P < 0.05). **(F)** Moderated-t log2 fold changes for axis genes (KRT5+ vs. KRT5-); stars denote BH-adjusted P < 0.05/0.01/0.001.

### 3.8 Cross-layer integration of cell-death-associated inflammatory remodeling

Effect directions between the SKOV3 KRT5^+/−^ contrast and the scRNA post-NACT EOC contrast were uncorrelated (Spearman r = −0.027, P = 0.0026; Figure 9A), consistent with these being orthogonal biological axes (cell identity vs. treatment remodeling). The direction matrix showed layer-specific effects: multiple candidates, including SPP1, MMP9 and CD44, were FDR-significant in TCGA SPP1-high tumors, while in the scRNA EOC and SKOV3 layers candidate effects were mostly nominal (Figure 9B). Among complete-case candidates, post-NACT EOC effects were modest and heterogeneous in direction (Figure 9C), reinforcing the negative pseudobulk result of Figure 6D. Univariate Cox models showed no program or SPP1 expression associated with overall survival at the nominal 5% level (all 95% CI crossed 1; E2F trended protective, P = 0.057; Figure 9D), so survival claims are not made. The positive SPP1-inflammatory program coupling observed in TCGA-OV (r = 0.63) was reproduced at sample level in the independent scRNA cohort (r = 0.71, n = 22; Figure 9E). A quantitative cross-layer summary confirmed strong positive SPP1-inflammatory coupling in TCGA-OV and scRNA samples and significant post-NACT increases in EOC hypoxia and VIM, while myeloid SPP1 and T/NK fraction changes remained non-significant (Figure 9F).

**FIGURE 9:**
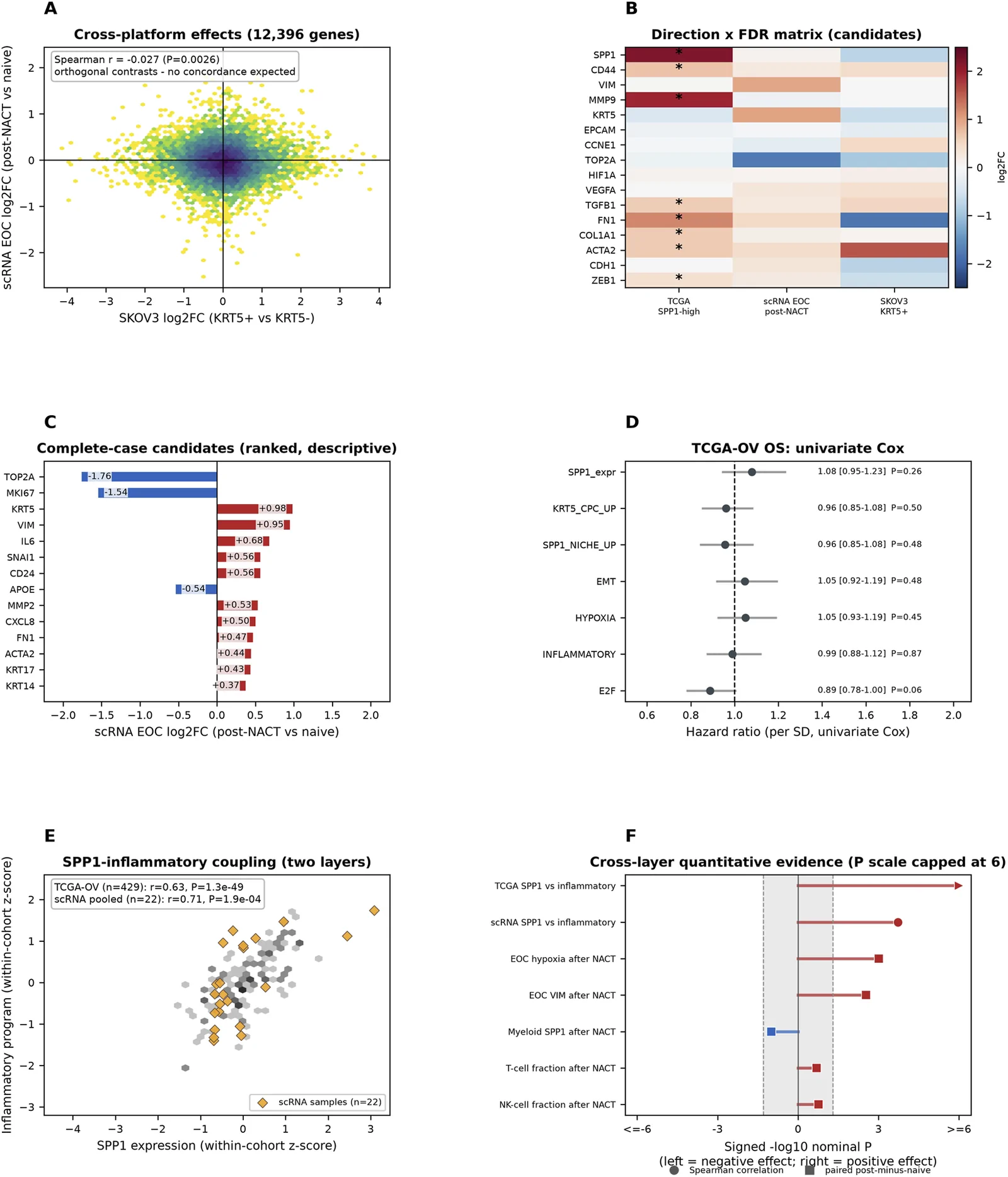
**(A)** Hexbin of per-gene effects: SKOV3 KRT5+ vs. KRT5- (x) and scRNA EOC post-NACT vs. naive (y); axes are independent native scales with zero reference lines. **(B)** Heatmap of per-layer log2 fold changes for candidate genes; asterisks mark FDR<0.05 within that layer; gray = not tested. **(C)** Complete-case candidate genes ranked by |scRNA EOC log2FC| (descriptive ranking; no composite significance is claimed). **(D)** Univariate Cox hazard ratios (per SD) with 95% CI for SPP1 and six programs; dashed line at HR = 1. **(E)** Within-cohort z-scored SPP1 expression versus inflammatory program: TCGA-OV hexbin with pooled scRNA samples overlaid as diamonds; Spearman r is annotated for both cohorts. **(F)** Quantitative cross-layer evidence summary. Points show signed -log10 nominal P (red/right = positive effect; blue/left = negative effect), capped at ± 6; circles denote Spearman correlations and squares paired effects. The shaded band is nominal P ≥ 0.05.

### 3.9 *In vitro* irradiation validation of cell-death-associated stress-response candidates

RT-qPCR validation was performed for four selected stress/inflammation-related candidate genes after *in vitro* irradiation. Relative to the 0-Gy control, the 6-Gy group showed significantly higher mean expression of SPP1 (2.31-fold), HIF1A (2.18-fold), VEGFA (2.47-fold), and KRT5 (2.05-fold), with P < 0.01 for each comparison (Figures 1A–D). These experimentally measured data support an acute irradiation-associated transcriptional response relevant to cell injury/death-associated inflammatory and hypoxia signaling, but they do not identify a specific regulated cell-death modality.

## 4 Discussion

This study integrates three complementary transcriptomic layers and an irradiation-based *in vitro* validation model to characterize the SPP1/KRT5 axis and treatment-associated microenvironment remodeling in HGSOC within a cell-death-associated inflammatory framework. The principal findings are that SPP1 tracks an inflammatory program in TCGA-OV, localizes predominantly to myeloid cells in the paired single-cell cohort, and remains positively coupled with inflammatory activity across independent bulk and single-cell datasets. In contrast, KRT5 expression is epithelial-restricted, and post-NACT remodeling is most clearly reflected by increased epithelial VIM and hypoxia-program activity rather than by a uniform shift across all lineages. Irradiation also increased SPP1, HIF1A, VEGFA, and KRT5 expression in SK-OV-3 cells, providing an orthogonal stress-response validation layer.

From a cell-death perspective, chemotherapy and ionizing radiation can engage multiple regulated cell-death programs, including apoptosis, necroptosis, pyroptosis, and ferroptosis, with distinct effects on membrane integrity, DAMP release, and immune activation (Galluzzi et al., 2018; Galluzzi et al., 2017). The present datasets were not designed to classify these modalities. Instead, our results capture downstream states that can accompany treatment-induced injury: SPP1-marked myeloid inflammation, epithelial hypoxia/EMT, and irradiation-responsive HIF1A/VEGFA/SPP1 expression. Thus, the data are most appropriately interpreted as supporting a death-associated inflammatory remodeling model rather than demonstrating a defined cell-death pathway.

The SPP1 findings are consistent with growing evidence that SPP1-positive tumor-associated macrophages represent a distinct microenvironmental state linked to immune suppression, matrix remodeling, and treatment resistance (Hou et al., 2025; Wang et al., 2025). In our data, however, SPP1 should not be interpreted as a stand-alone prognostic marker: the TCGA median-split analysis showed no significant overall-survival difference, and univariate Cox models for SPP1 and the analyzed programs did not reach the nominal 5% significance level. The reproducible SPP1-inflammatory correlation therefore supports a biological association with inflammatory microenvironmental activity rather than a validated survival biomarker.

The paired GSE165897 analysis further indicates that chemotherapy-associated remodeling is lineage-specific. Epithelial VIM and hypoxia-program scores increased after NACT, whereas myeloid SPP1 showed only a non-significant downward trend. The original GSE165897 study similarly described chemotherapy-associated stress-state remodeling in malignant and stromal compartments (Zhang et al., 2022). Our observations extend that framework by showing that epithelial hypoxia/mesenchymal changes and myeloid SPP1 behavior need not move in the same direction. Because the present analyses are observational, these patterns may reflect shared treatment-associated upstream programs rather than direct regulatory relationships between the measured genes or cell states.

The KRT5-sorted SKOV3 dataset provides a separate cell-state axis rather than a direct model of post-NACT change. The reciprocal enrichment of the KRT5_CPC_UP and SPP1_NICHE_UP signatures is expected because these exploratory signatures were derived from the same KRT5+/KRT5-contrast and therefore does not constitute independent validation. Recent experimental work using this system has shown that KRT5-positive HGSOC cells have cancer-propagating properties and that KRT5-negative cells can provide an SPP1/osteopontin-rich supportive niche (Bidarimath et al., 2026). In our patient single-cell data, KRT5 itself remained epithelial-restricted, but the cell-line-derived KRT5 program scored highest in myeloid cells, underscoring that a signature derived from a cell-line contrast can be influenced by genes expressed in different compartments when transferred to complex tumors.

Cross-layer comparison also argues against collapsing the different datasets into a single composite biological axis. Gene-level effects in the SKOV3 KRT5+/KRT5- contrast and the epithelial post-NACT contrast were essentially uncorrelated, consistent with cell identity and treatment remodeling representing distinct sources of variation. By contrast, the SPP1-inflammatory relationship was reproduced across TCGA-OV and the single-cell cohort. These findings support using cross-layer concordance selectively, where the same biological relationship is observed in independent contexts, rather than treating all signatures as interchangeable.

Accordingly, the translational implications should remain cautious. The present data nominate SPP1-associated myeloid inflammation, KRT5-associated epithelial heterogeneity, and hypoxia-linked stress responses as features at the interface of treatment injury, cell-death-associated signaling, and tumor-microenvironment remodeling. They do not establish direct regulation, a specific death modality, treatment sensitivity, or therapeutic efficacy. Functional perturbation, direct measurement of death-pathway execution and DAMP release, independent patient cohorts, and preferably spatial or longitudinal validation will be required before these associations can be used for biomarker development or precision targeting.

### 4.1 Limitations

Several limitations should be considered. First, TCGA-OV is a cross-sectional bulk cohort and cannot establish within-patient treatment-induced changes. Second, the paired scRNA-seq analysis is based on a single public dataset comprising 11 patients; although sample-level paired tests were used for inference, the cohort remains small and requires external replication. Third, GSE316118 contains only three KRT5+ and three KRT5-libraries, and the KRT5_CPC_UP and SPP1_NICHE_UP signatures were derived from that same contrast; their reciprocal separation is therefore exploratory and not independent validation. Fourth, bulk TCGA-OV, patient scRNA-seq, and SKOV3 data were analyzed as separate layers rather than directly batch-integrated because they represent different assay types and biological contexts. Fifth, gene-gene and program-level correlations are associative and may arise from shared upstream states such as inflammation, hypoxia, or treatment stress rather than direct co-regulation. Sixth, no direct measurements of regulated cell-death execution or DAMP release were performed; modality-specific assays such as lipid peroxidation/GPX4 for ferroptosis, cleaved GSDMD/caspase-1 for pyroptosis, phospho-MLKL/RIPK3 for necroptosis, or extracellular HMGB1 would be required to support such assignments (Galluzzi et al., 2018; Galluzzi et al., 2017). Finally, no genetic perturbation or spatial validation was performed, limiting causal interpretation and the immediate translational significance of the identified relationships.

## 5 Conclusion

By integrating TCGA-OV bulk transcriptomics, paired pre/post-NACT single-cell data, KRT5-sorted SKOV3 transcriptomes, and irradiation-based RT-qPCR validation, this study supports a cell-death-associated inflammatory remodeling framework in HGSOC. SPP1 consistently tracked inflammatory activity across bulk and single-cell contexts, whereas post-NACT remodeling was characterized most clearly by increased epithelial hypoxia and VIM expression; irradiation further induced SPP1, HIF1A, VEGFA, and KRT5 *in vitro*. The KRT5+/KRT5-axis captured a distinct exploratory cell-state contrast rather than the same treatment-response process. Together, these findings link the biological context of treatment-related cellular injury/death with myeloid inflammation and epithelial stress states without establishing any particular regulated cell-death modality. Direct measurements of death execution and DAMP release will be required to test this framework mechanistically.

## Data Availability

The datasets presented in this study can be found in online repositories. The names of the repository/repositories and accession number(s) can be found in the article/Supplementary Material.
